# Phosphate binders as a cause of hypothyroidism in dialysis patients: practical indications from a review of the literature

**DOI:** 10.1186/s12882-018-0947-9

**Published:** 2018-07-02

**Authors:** Emanuela Cataldo, Valeria Columbano, Louise Nielsen, Lurlynis Gendrot, Bianca Covella, Giorgina Barbara Piccoli

**Affiliations:** 1 Néphrologie Centre Hospitalier le Mans, Avenue Roubillard, 72000, Le Mans, France; 2 Nefrologia, Università della Campania “Luigi Valvitelli”, Naples, Italy; 3 Nefrologia, Università degli Studi di Bari “Aldo Moro”, Bari, Italy; 4 Dipartimento di Scienze Cliniche e Biologiche, Università di Torino, Turin, Italy

**Keywords:** Hemodialysis, Polypharmacy, Levothyroxine, Phosphate binders, Fatigue

## Abstract

**Background:**

Although fatigue is common in dialysis patients, polypharmacy is seldom listed among its causes. In this report, we describe a dialysis patient who developed severe fatigue due to pharmacological interaction between two commonly prescribed drugs, phosphate binders and levothyroxine.

**Case Presentation:**

A 65-year old woman, on dialysis for 17 years, complained of fatigue (weight 54 Kg, height 1.55 m, BMI: 23 Kg/m2; malnutrition inflammation index: 10; Charlson index 9). She had been treated with lithium for about 20 years. A heavy smoker, she was obese and diabetic when young, but stopped treatment after weight loss. She had undergone thyroidectomy for papillary carcinoma, left hemicolectomy for colon adenocarcinoma, left quadrantectomy followed by radiotherapy for ductal mammary adenocarcinoma, subtotal parathyroidectomy for tertiary hyperparathyroidism. At the time of this report, she was on thrice-weekly hemodiafiltration (Daugirdas 2 Kt/V: 1.6–1.8). Her recent treatment included spironolactone, amlodipine, perindopril, valproate, lamotrigine, levothyroxine, vitamin D, calcium carbonate, sodium polystyrene and sevelamer. After she questioned her doctor about whether her fatigue might be the result of a drug interaction, levothyroxine interference was identified (TSH, previously normal, increased to 13.07 mU/L, after increasing sevelamer dose, and normalized after change of drug schedule).

Literature review: only 5 relevant papers on levothyroxine and phosphate binders on dialysis were found on Pubmed and EMBASE (out of 351 titles retrieved). Information was therefore inferred from studies in normal volunteers or in other diseases.

**Discussion and conclusions:**

Our case differs from other reports on lower TSH at diagnosis, underlining the need for awareness of the importance of early diagnosis. Integrating the scant literature on dialysis patients with data available in the general population, some working conclusions can be reached: while all phosphate binders potentially interfere with levothyroxine absorption, interference seems to be highest for sevelamer; interference is limited but not excluded by increasing the intervals between drugs; morning fast is usually indicated but, when clashing with the timing of other drugs, a bedtime dose and liquid preparations may be indicated. In the absence of an agreed control schedule, our case supports close monitoring of TSH (1–3 months if unstable, twice-yearly in stable patients).

## Background

Fatigue is a debilitating symptom very often experienced by patients undergoing dialysis [1–3].

In this context, fatigue can have a variety of causes, such as anemia, the malnutrition inflammatory syndrome, nutritional deficits, insufficient dialysis, fluid overload, depression and chronic pain [1–7]. Despite the increasing interest in reducing the number of drugs taken by chronic dialysis patients, polypharmacy is seldom listed among the common causes of fatigue.

In this report, we describe a patient on chronic dialysis who developed severe fatigue due to pharmacological interaction between two drugs commonly prescribed for dialysis patients: phosphate binders and thyroid hormone replacement therapy.

Patients with end-stage renal disease (ESRD) exhibit various changes in thyroid function: according to some studies, between 10 and 25% of dialysis patients have some kind of thyroid derangement [8–12]. Hypothyroidism accounts for most of these cases [8–12].

Levothyroxine is therefore commonly used to treat ESRD patients (estimated as used by 2 to 10% of these patients) and, while it is well known that many drugs and foods interfere with its absorption, no study has analyzed the multiple potential interferences in patients on chronic hemodialysis, who are typically on a complex polypharmacy [13, 14].

Hence, the case described in this study provided an opportunity to review the literature and draw some practical conclusions regarding the interferences between levothyroxine and phosphate binders, the drugs most commonly used by dialysis patients. The description of the case is followed by a systematic review of the literature on the interference between levothyroxine and phosphate binders in dialysis patients, integrated wherever possible by data obtained on non-dialysis patients, making it possible to offer a reasoned guide to prescription, while highlighting the limits of the current evidence.

## Case Presentation

A 65-year old woman, on dialysis for 17 years, told her doctor she was exhausted and that she thought it was because she was taking too many drugs. The patient is an intelligent, independent, acculturated woman who lives alone in the French countryside.

Her medical history is complex: she was treated with lithium for about 20 years (from age 20 to 40). This was discontinued after she developed CKD; in recent years, under treatment with valproic acid and lamotrigine, her psychophysical balance has been good,.

She started smoking when she was 19 years old (30 cigarettes/day) and developed a smoke-related chronic obstructive pulmonary disease. She was obese in early adulthood; arterial hypertension was diagnosed at age 30 and type 2 diabetes at age 32, treated using oral hypoglycemic drugs, but she eventually lost about 20 kg, making it possible for antidiabetic drugs to be discontinued. She underwent total thyroidectomy for papillary carcinoma at age 41, and started levothyroxine therapy afterwards. Due to a progressive worsening of the kidney function she started hemodialysis at age 50. Her kidney disease was probably multifactorial (hypertension, diabetes, obesity, heavy smoking, lithium therapy).

Seven years after dialysis start, she underwent left hemicolectomy for colon adenocarcinoma, and two years later, left quadrantectomy followed by radiotherapy for ductal mammary adenocarcinoma. She underwent subtotal parathyroidectomy for severe tertiary hyperparathyroidism at age 62. Due to the presence of severe scoliosis, and the development of peripheral neuropathy, she uses painkillers regularly.

At the time of the present report, she was on thriceweekly hemodiafiltration, with good dialysis tolerance and high dialysis efficiency (Daugirdas 2 Kt/V: 1.6–1.8).

Her most recent treatment included antihypertensive drugs (spironolactone 100 mg, amlodipine 20 mg, perindopril 2.5 mg), antipsychotic drugs (valproic acid 600 mg, lamotrigine 100 mg), thyroid hormone (levothyroxine 150 μg), vitamin D, bicarbonate and calcium supplements (calcium carbonate 1 g, sodium bicarbonate 500 mg, vitamin D 25-OH 100,000 UI once a month), potassium and phosphate binders (sodium polystyrene sulphonate, on non-dialysis days and sevelamer 2.4 g per day), darbopoietin 20 μcg once weekly.

The clinical examination revealed a woman with good psychophysical balance, a moderate impairment in nutritional status, and a severe comorbidity burden (weight 54 Kg, height 155, BMI: 23 Kg/m2; subjective global assessment: B; malnutrition inflammation index: 10; Charlson index: 9). Apart from signs of chronic bronchitis, and an aortic 2/6 heart bruit, the clinical examination was unremarkable; arterial blood pressure was 150/90 mmHg, with mild orthostatic hypotension (135/80 mmHg); heart rate was 68 bpm. The most recent biochemical results are reported in Table 1.

**Table 1:** Main biochemical data in our patient

	Month 1	Month 2	Diagnosis (month 3)
Hemoglobin g/dl	14.5	11.5	11.5
Urea predialysis mg/dl	159.03	160.84	131.92
Kt/V	1.78	1.59	1.55
Creatinine mg/dl	7.96	7.85	6.33
Na mmol/l	142	145	145
K mmol/l	4.8	4.7	4.5
Colesterol mg/dl	200	164	–
Albumin g/l	34	34	34
Total proteins g/l	73	70	72
CRP mg/l	10	< 4	< 4
BNP pg/ml	242	202	340
Transferrin mg/dl	206	206	225
PTH ng/l	92	181	199
Vitamin D μg/l	51	52	64
Ca mg/dl	10.12	8.24	8.12
Phosphate mg/dl	9.64	9.11	6.75

Legend: *BNP* blood natriuretic peptide; *CRP* C- reactive protein; *PTH* parathyroid hormone

The patient complained of severe fatigue, which had recently increased, and asked her doctor if he thought this could be the result of taking too many drugs.

In fact we thought our patient was right about pharmacologic interference, and felt that levothyroxine was the most likely candidate. A biochemical control disclosed a relevant increase in TSH (13.07 mU/L), as compared with her previous routine twice-yearly control (4.14 mU/L).

In retrospect, the levothyroxine dose was already high for a woman weighing about 55 Kg, and a reduction in the absorption of levothyroxine should already have been suspected.

Three of her chronic drugs display potential interference with levothyroxine: calcium carbonate, sevelamer and kayexelate. Since her sevelamer dose had recently been increased from 800 mg once daily to 800 mg 3 times per day, this long-scting phosphate binder was the most likely candidate. In keeping with this hypothesis, one month after discontinuation of sevelamer and modification of the timing of levothyroxine treatment (in the morning after night fasting) TSH was once more in the normal range. In the three months that followed, her levothyroxine dose was reduced to 100 micrograms per day. A further attempt to reintroduce sevelamer, taken at least 6 h after levothyroxine, led to a new increase in TSH (TSH 12.66 mUI/l), once more corrected by discontinuation of sevelamer.

### Systematic review of the literature

**Fig. 1:**
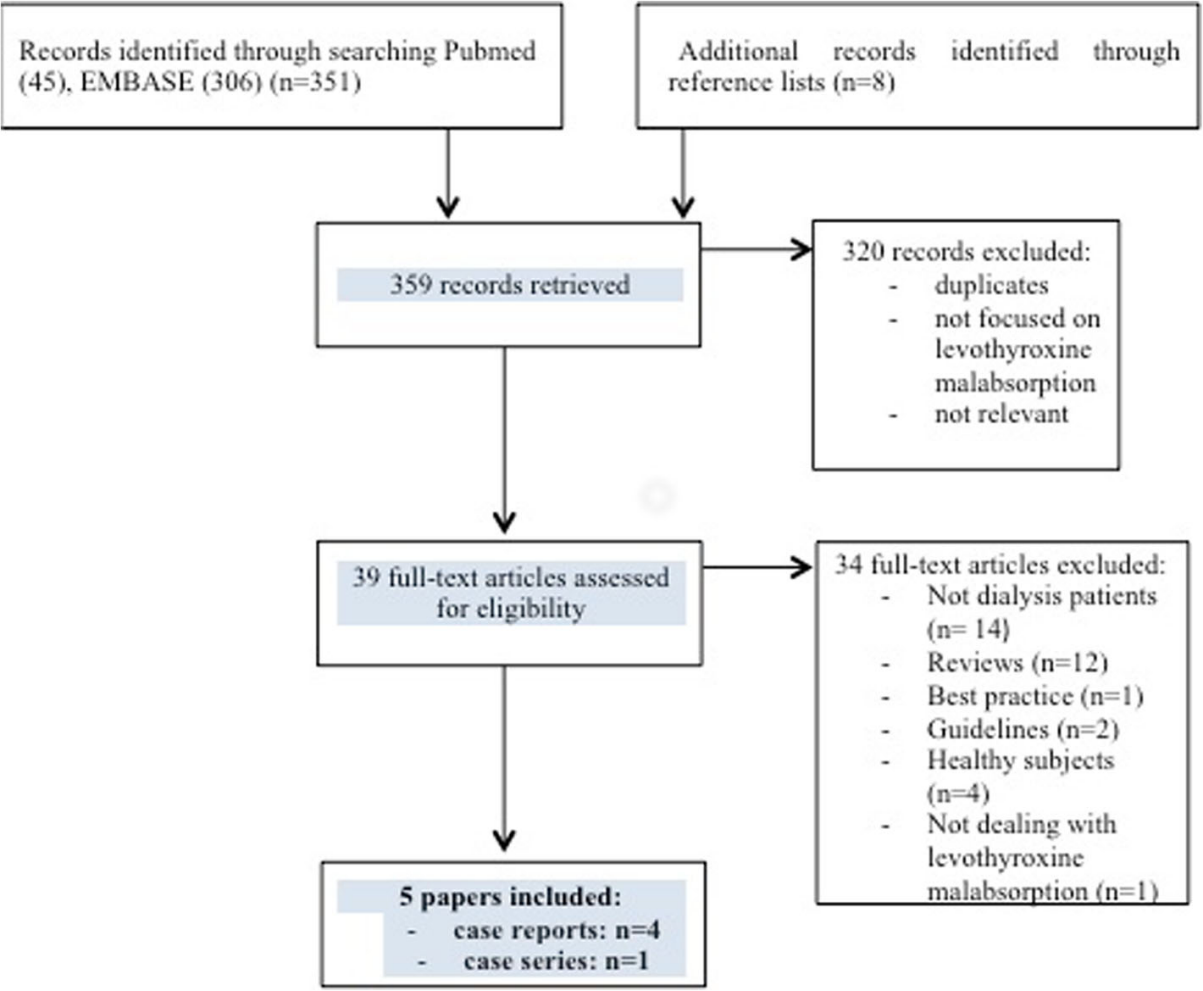
Search strategy and selection of papers

Pubmed and EMBASE were explored (start to February 15th 2018) with the aim of retrieving papers related to dialysis, levothyroxine and phosphate binders. The following terms were employed: (a) dialysis, hemodialysis, hemodiafiltration, renal replacement therapy; (b) phosphate binder(s), sevelamer, calcium carbonate, calcium acetate, aluminum hydroxide, lanthanum carbonate; (c) levothyroxine, thyroid hormone replacement therapy. Due to the low number of papers retrieved by this search, a further search was performed combining (b) and (c). The searches, paper selection and data extraction were performed in duplicate (EC and VC); a further search on Google med did not lead to further papers. Discrepancies were resolved by discussion with a third party (GBP). A manual search of the reference lists from identified articles was done to identify additional articles. The search strategy and flow-chart is reported in Fig. 1.

Given the small number of papers retrieved, and their high heterogeneity, a meta-analysis was not performed, and the data were narratively discussed, in relation to other sources of information (data obtained in healthy volunteers, or in other diseases).

## Discussion and conclusions

Many physiological and pathological conditions can alter levothyroxine absorption [15–17].

In addition to gastrointestinal diseases, from gastritis to helicobacter infection, inflammatory bowel disease and intestinal infections, and malabsorption, several foods and beverages (including dietary fibers, soybeans, herbal remedies, papaya, grapefruit, and coffee) affect the absorption of levothyroxine [15].

However, in patients on polypharmacy, pharmacological interferences are the major cause of levothyroxine malabsorption [15–18]. Several drugs are listed; the effect is usually higher when taken simultaneously or less than one hour before or after taking levothyroxine. The potential list in dialysis patients is long, and includes gastro-protective agents, antibiotics, bile acid sequestrants, oral iron, potassium binders (sodium polystyrene sulphonate) and, most importantly, phosphate binders [15–18].

Phosphate binders are probably the drug most widely used by dialysis patients and virtually all phosphate binders interfere with levothyroxine absorption. These include aluminum hydroxide, calcium salts (calcium carbonate, calcium citrate and calcium acetate), sevelamer, magnesium hydroxide, lantanum carbonate and sucrofferic oxyhydroxide [19–32].

The mechanism of phosphate binding is different. Some agents use ionic interactions to attract, bind and precipitate phosphate; the insoluble phosphate compound is ultimately excreted in the feces.

Aluminum hydroxide, calcium salts, magnesium hydroxide, and lantanum carbonate are inorganic salts which release ions that trap dietary phosphate in the gut. Sucrofferic oxyhydroxide, instead, is a new compound containing an iron (III)-oxyhydroxide core which binds phosphate in the intestinal lumen [29, 30]. Sevelamer is different from the other phosphate binders because as it is composed of a cationic hydrogel with multiple amine groups which become protonated in the gastrointestinal tract and bind with anionic phosphate and other anionic substances, including certain drugs, interfering with their absorption [32]. This may explain the higher number of pharmacological interactions described with sevelamer.

While we know that pharmacokinetics and binding avidity often differ, most of the available data were obtained in cohorts that are clinically different from dialysis patients. For instance, the interference between calcium supplements and levothyroxine absorption has mainly been studied in post-menopausal women [21, 22, 24–27]. Moreover, in some studies other interfering drugs were excluded [16, 21, 23, 30, 31, 33]. Data on the interference between levothyroxine absorption and the phosphate binders specifically used in dialysis patients are derived principally from studies using healthy volunteers [16, 23, 30, 33]. Therefore, the indications on treatment modalities were obtained in settings that are profoundly different from the complex polypharmacy of dialysis patients, a population in which malnutrition, malabsorption, gastropathy and diabetic neuropathy often play a relevant role in modifying absorption [15, 34–36].

Our review of the literature, which focused on the evidence specifically related to dialysis patients, retrieved only 5 papers, of which 4 were case reports and 1 was a case series (Fig. 1, Table 2). Based on this limited body of evidence, interference seems to be higher for sevelamer than for calcium salts, lanthanum and preparations containing aluminum [28, 31, 37–39].

**Table 2:** Papers on levothyroxine malabsorption in dialysis patients taking phosphate binders, retrieved after a systematic review of the literature

Author (reference)	n	Study design	Age	RRT Vintage (years)	Phosphate binder(s)	TSH mU/L	Associated drugs	Therapeutic measures
Iovino 2014 [21]	1	Case report	26	2	Sevelamer	650	No potentially interfering drugs	Sevelamer at least 4 h after laevothyroxyne
Wong, 2012 [32]	1	Case report	30	12	Sevelamer	~ 600	Not reported	Switch to sublingual levothyroxine. Sevelamer far from levothyroxine
Granata, 2011 [30]	1	Case report	55	3	Sevelamer	153	Ramipril, Pantoprazole	Levothyroxine 2 h after dinner
Arnadottir 2007 [31]	1	Case report	62	NA	Sevelamer	297	Amlodipine, Enalapril, Esomeprazol, Paracetamol, Vitamins	Levothyroxine at night, at least 4 h after other drugs
Diskin, 2007 [23]	67	Retro-spective study	74.74 (mean)	NA	Calcium carbonate, Calcium acetate, Sevelamer	**	Patients taking interfering drugs were excluded	Changing timing of levothyroxine or switching to calcium acetate

Legend/NA/not available

Note: ** TSH levels (mean +/− SD): 3.92+/− 7.83 (calcium acetate); 23.7974+/− 19.50 (calcium carbonate); 20.2908+/− 30.83 (sevelamer)

The one large observational study of hemodialysis patients on levothyroxine and on different phosphate binders involved 67 individuals. It found higher TSH levels in patients on calcium carbonate and sevelamer, than in those being treated with calcium acetate [31]. However, it excluded all other “potentially interfering drugs”, and its lack of information on associated therapies in the different groups of patients represents a limit of this important study [31].

Another limit of the current literature is that it does not provide evidence on the dose at which the interaction is most significant, nor it gives insight on a threshold effect. In the absence of this information, we hold that the effect is non dose dependent.

**Fig. 2:**
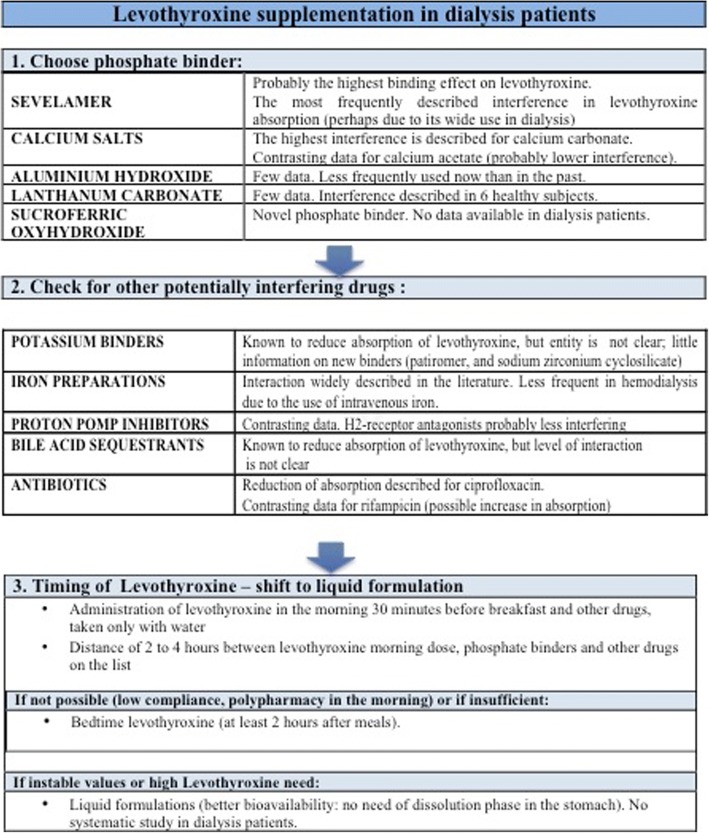
Levothyroxine supplementation in dialysis patients: practical insights

Our case differs from the other cases reported in literature in the level of TSH at diagnosis, which was over 100 in the other four case reports. In fact, in many settings, TSH is not routinely checked in dialysis patients, and, due to the clinical mimicry of hypothyroidism and dialysis-related symptoms, first of all fatigue, diagnosis may be delayed. While suggesting systematic TSH testing in all dialysis patients is beyond the scope of this report, we have tried to show that the available evidence supports its clinical management in dialysis patients on levothyroxine. Figure 2 and Table 3 summarize the answers to four questions: Which phosphate binders should be prescribed? At what time intervals should they be taken? Are there alternatives? How often should patients be monitored??

**Table 3:** Indications for controls

Controls over time:	
No agreed indication	
Monitoring TSH and fT4 at least every 3 months in patients on one phosphate binder + one other potentially interfering drug (on the basis of the high levels found in the previous studies, and in ours)	
Monthly monitoring if unstable TSH or if new potentially interfering drugs, change in dose of phosphate binder, or more than 2 potentially interfering drugs (importance of early diagnosis)	
Reduce frequency when there has been long-term stability, and no change in treatment (probably wise to monitor patient at least twice a year)	

The first point is the choice of the phosphate binder. Although sevelamer seems to be the one with the highest degree of interference, in the absence of precise data, all phosphate binders should be considered to be potentially capable of interfering with levothyroxine absorption [28, 31, 37–39].

The second point regards the timing of levothyroxine administration: the standard recommendation is to take it at least 30 min before breakfast. However, this may conflict with the complex polypharmacy commonly prescribed in dialysis patients. Delayed gastric emptying, linked to diabetes or to uremia, may also play a role in retarding gastric emptying and reducing absorption [35, 36]. In line with what is known, a phosphate binder should be taken at an interval of at least two hours from levothyroxine, keeping the interval between LT4 and binding agents as constant as possible [15]. An alternative could be bedtime administration of levothyroxine, which may be favorable in particular in patients who dine early and consume their largest meal at noon, thus minimizing interferences with food [40, 41].

Several studies have shown better pharmacokinetics and bioavailability of liquid formulations and of soft gel capsules, whose faster absorption is less affected by food and drug interactions [42–45]. However, the advantage has to be balanced against the risk of errors, which is higher with drops than with pills. Furthermore, the cost of liquid formulations is higher (for example, in Italy, 0.331 euros/day versus 0.0578 euros/day for tablets) [42].

The last question is how often patients need to be monitored. Given the absence of data on the effect of simultaneous administration of several potentially interfering drugs, we suggest a very cautious approach, in particular in potentially interfering polypharmacy, and in case of treatment or posology changes (Table 3).

In summary, this case points out the importance of pharmacological interaction in dialysis patients and suggests paying particular attention to levothyroxine in the context of polypharmacy. Our review was focused on phosphate binders, which probably represent the most commonly used drugs in dialysis patients, while other frequently used drugs, including proton-pump inhibitors and potassium binders can reduce levothyroxine absorption [15–18].

In the absence of studies addressed to the modulation of levothyroxine absorption by different combination of commonly used drugs in dialysis patients, our case provides evidence that TSH should be closely monitored at regular intervals in dialysis patients on thyroid hormone replacement therapy.

## Abbreviations

BMI: Body mass index
CKD: Chronic kidney disease
ESRD: End-stage renal disease
TSH: Thyroid stimulating hormone
